# A New Medical Society

**Published:** 1886-06-20

**Authors:** C. C. Hart

**Affiliations:** Secretary


### A NEW MEDICAL SOCIETY.

At a meeting of DeKalb County Physicians held in Decatur, Ga., May 29, 1886, on motion Dr. J. L. Hamilton was called to the Chair and Dr. C. C. Hart requested to act as Secretary. Dr. Hamilton explained the object of the meeting to be the organization of a medical society.

On motion Drs. R. C. Word, A. S. Mayson and A. L. Jones were appointed a committee to draft a Constitution and By-Laws for the government of the Society; said Constitution and By-Laws to be submitted for adoption at the next meeting. The above mentioned committee was also instructed to invite regular physicians of Gwinnett, Rockdale and DeKalb counties to be present at the next meeting to consider the propriety of organizing a tri-county association. On motion it was agreed to hold the next meeting at Stone Mountain, at 10 a. m., on the third Saturday, June 19, 1886.

C. C. HART, Secretary.

